# Ether

**Published:** 1866-08

**Authors:** 


Ether.—A wealthy gentleman of Boston has placed a sum of money in the hands of an architect, for erecting a monument in that city to commemorate the discovery of the anæsthetic properties of Ether.

